# Advances About Immunoinflammatory Pathogenesis and Treatment in Diabetic Peripheral Neuropathy

**DOI:** 10.3389/fphar.2021.748193

**Published:** 2021-10-04

**Authors:** Tianyu Xue, Xin Zhang, Yiwen Xing, Shuhan Liu, Lijun Zhang, Xun Wang, Min Yu

**Affiliations:** ^1^ Department of Neurology, The Third People’s Hospital of Dalian, Non-Directly Affiliated Hospital of Dalian Medical University, Dalian, China; ^2^ Department of Ophthalmology, The Third People’s Hospital of Dalian, Non-Directly Affiliated Hospital of Dalian Medical University, Dalian, China; ^3^ Department of Neurosurgery, The Third People’s Hospital of Dalian, Non-Directly Affiliated Hospital of Dalian Medical University, Dalian, China

**Keywords:** diabetic peripheral neuropathy, immune inflammation, therapy, immune cells, pathogenesis

## Abstract

Most diabetic patients develop diabetic peripheral neuropathy (DPN). DPN is related to the increase of inflammatory cells in peripheral nerves, abnormal cytokine expression, oxidative stress, ischemia ,and pro-inflammatory changes in bone marrow. We summarized the progress of immune-inflammatory mechanism and treatment of DPN in recent years. Immune inflammatory mechanisms include TNF-α, HSPs, PARP, other inflammatory factors, and the effect of immune cells on DPN. Treatment includes tricyclic antidepressants and other drug therapy, immune and molecular therapy, and non-drug therapy such as exercise therapy, electrotherapy, acupuncture, and moxibustion. The pathogenesis of DPN is complex. In addition to strictly controlling blood glucose, its treatment should also start from other ways, explore more effective and specific treatment schemes for various causes of DPN, and find new targets for treatment will be the direction of developing DPN therapeutic drugs in the future.

## Introduction

**FIGURE1:**
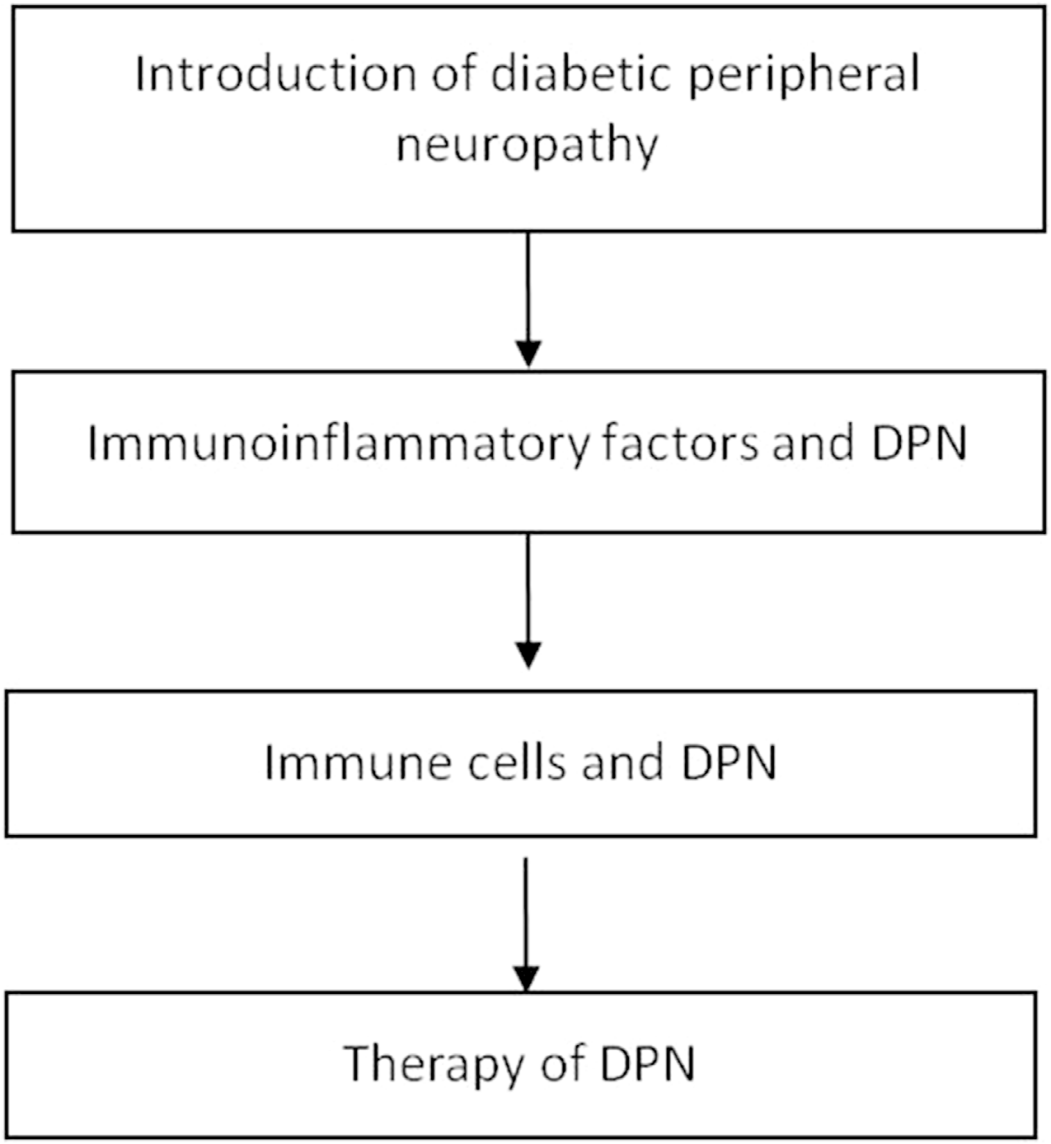
Schematic flowcharts. Schematic flowcharts of the article.

The prevalence of diabetes mellitus (DM) increased with age. DM is present in 19.9% of 65–79-year-olds (Saeedi et al., 2019). Diabetic peripheral neuropathy (DPN) is one of the common chronic complication of diabetes mellitus. Its Clinical Syndrome can be dysfunction of peripheral neuropathy, influencing the quality of life in the dabetic patients. DPN is a length-dependent sensory axonal lesion that usually the first manifestation is the sensory disturbance, pain, numbness or loss of balance (Edwards et al., 2008). DPN leads to the decline of patient’s quality of life. Studies have shown that although blood glucose control can reduce the incidence of peripheral neuropathy in type 1 diabetes, it will also increase the incidence of hypoglycemia, and aggressive blood glucose control can not reduce the incidence of peripheral neuropathy in type 2 diabetes (Group 1993; Boussageon et al., 2011; Callaghan et al., 2012a). Moreover, although neuropathy associated with T1DM and T2DM has been classified into the same category, we know that the pathogenesis of these two diseases is very different (Callaghan et al., 2012b; Eid Sas et al., 2019). It is essential to study the pathogenesis of DPN. In the past, most studies on DPN were limited to metabolism, genetic mechanism, and the effect of hypoglycemic drugs (Ferland-Mccollough et al., 2016; Sztanek et al., 2016; De et al., 2017; Stino and Smith, 2017; Jamwal et al., 2018). Previous drug trials mainly focused on antioxidants (Ziegler et al., 1999; Ametov et al., 2003; Ziegler et al., 2006), aldose reductase inhibitors (Hotta et al., 2006; Bril et al., 2009; Ramirez and Borja, 2012), neurotrophic factors (Apfel 2002; Decroli et al., 2019), GABA analogs (Ermis et al., 2010), cell metabolic agonists (Evans et al., 2008; Dan, Movsesyan et al., 2009) and vasodilators (Yuen et al., 2002). These drugs have limited effect in the clinical treatment of DPN associated with T1DM and T2DM (Coert, J. et al., 2016). However, with the further study of DM, the mechanism of immune inflammation is related to the pathogenesis of DPN. This review mainly focuses on the immune-inflammatory mechanism of DPN. (Figure 1).

## Immunoinflammatory Factors and Diabetic Peripheral Neuropathy

### Tumor Necrosis Factor-α

**FIGURE2:**
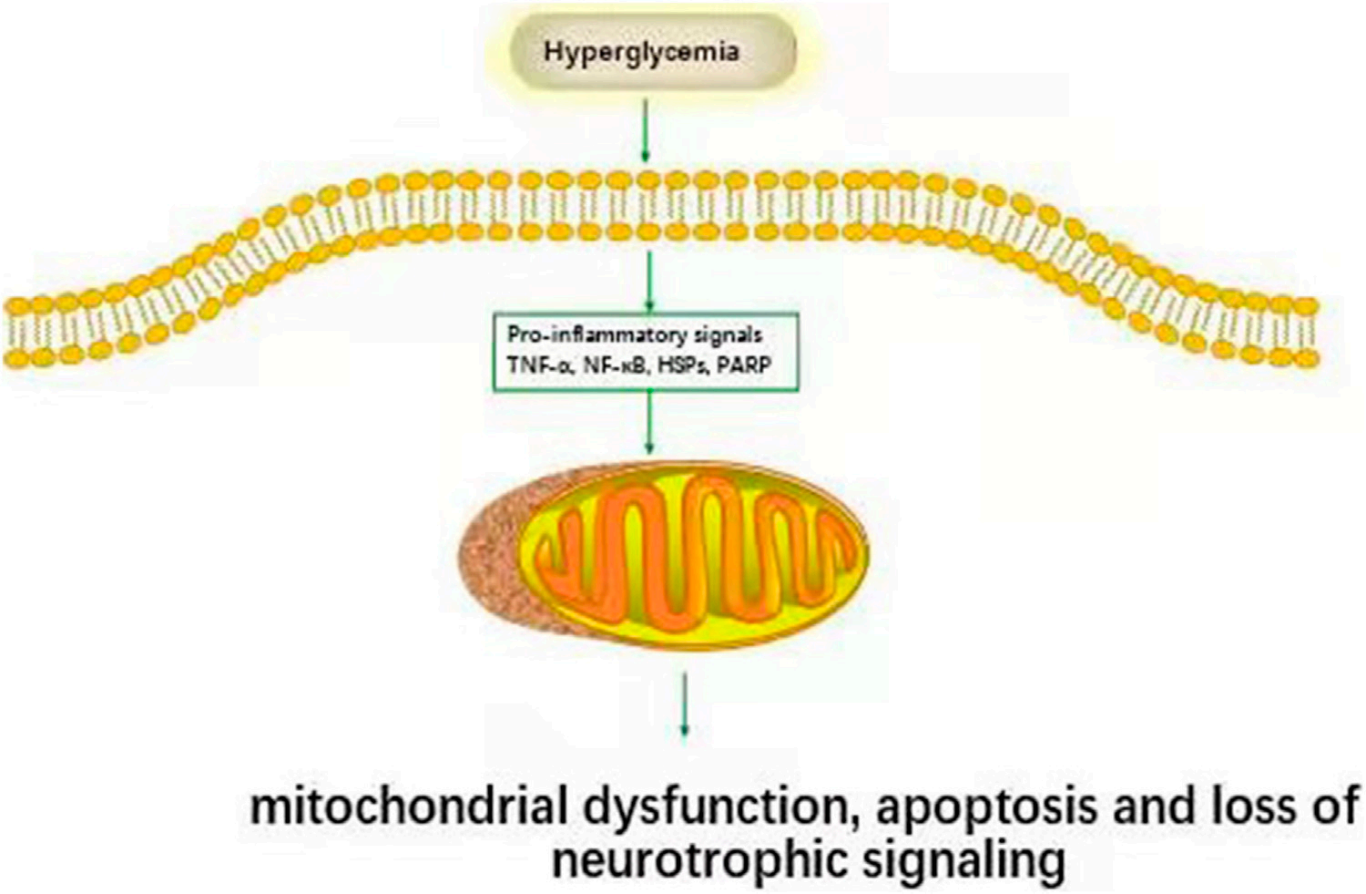
Inflammatory pathways associated with DPN. Hyperglycemia activates inflammatory pathways (TNF-α, NF-κB, HSPs, PARP et al.) and causes cell damage.

Figure 2 Hyperglycemia has been shown to activate tumor necrosis factor-α (TNF-α) secretion from immune cells. TNF-α causes nerve demyelination and stimulates monocytes and endothelial cells to secrete inflammatory substances, further increase nerve damage. DPN patient’s serum tumor necrosis factor-α (TNF-α) level was significantly higher than that of patients without DPN and normal controls. The risk of DPN in diabetic patients with elevated TNF-α was 2.594 times that of regular TNF-α patients (Mu et al., 2017). The serum tumor TNF-α of DPN patients was higher than patients without DPN and normal controls (Duksal et al., 2016). The most conclusive finding is that streptozocin (STZ) induced diabetes TNF- α-/-mice will not develop DPN like diabetic mice (Yamakawa et al., 2011). Hussain et al. found that TNF-α was negatively correlated with nerve conduction velocity (Hussain et al., 2013). Shi et al. showed that compared with untreated DPN, recombinant human TNF-α receptor antibody fusion protein inhibited TNF-α in STZ induced diabetic rats, partially improving motor nerve conduction velocity (MCV) and sensory nerve conduction velocity (SCV), increasing the expression of myelin basic protein (MBP), and preventing myelin and neural structural abnormalities (Shi et al., 2013).

Saleh et al. (2011) showed that the expression of TNF-α and interleukin-6 (IL-6) in the dorsal root ganglia (DRG) induced by STZ was downregulated in 2 and 5 months after induction, but it was found in the nerve. In addition, we also found that TNF-α induced neurite growth via the nuclear factor kappa-B (NF-κB) pathway in sensory neurons *in vitro*. However, the growth of this neurite was markedly impaired in sensory neurons of diabetic rats (A, A et al., 2011). It is concluded that the decrease of TNF-α in DRG may lead to the repair and regeneration of DPN -related nerve injury. Grosick et al. studied the response of macrophage phenotypes to high glucose and typical glucose environments. We found that in a high glucose environment, macrophages stimulated by LPS produced high levels of TNF-α and human macrophage chemoattractant protein-1 (MCP-1), but the effect of IL-6 was not so strong. However, lipopolysaccharide (LPS) stimulation did not significantly increase MCP-1 or TNF-α under normal glucose conditions compared with unstimulated macrophages. In addition, macrophages exposed to LPS and IL-10 maintained high TNF-α expression under high glucose conditions, while macrophages exposed to average glucose maintained high TNF-α expression; Only LPS stimulation could increase the secretion of TNF-α (Grosick et al., 2018).

### Heat Shock ProteinS

Heat shock protein (HSP) is a chaperone protein synthesized by the body under stress (Sun and Li, 2003). HSP regulates the biological activities of various proteins under non-stress conditions, including regulating DNA replication, gene transcription, protein transfer of subcellular structure, cell signal transduction, immune response, growth, development, and apoptosis (Takahashi et al., 2003). Korngut et al. demonstrated that STZ induced diabetic mice overexpressed HSP27 (Korngut et al., 2012). Diabetic animals with high expression of HSP27 had axonal terminal density and mechanical sensitivity similar to those in the non-diabetic control group, which was in contrast with those in the diabetic non-transgenic control group. In addition, SCV in diabetic mice with high expression of HSP27 did not slow down compared with diabetic non-transgenic mice, but MCV was similar to that in diabetic mice. This study also showed that over expression of HSP27 in diabetic mice decreased receptor for advanced glycation endproducts (RAGE) positive sensory neurons compared with diabetic non transgenic-mice. It is worth further noting that compared with non-transgenic mice, the expression of activated caspase-3 as a cytotoxic marker of diabetes decreased in HSP27 overexpression mice. Elevated levels of HSP70 may be related to insulin resistance in type 2 diabetic patients. The number of endothelial progenitor cells decreased, and their function was impaired in T2DM patients, positively correlated with atherosclerotic cardiovascular events. These factors are closely related to complications such as diabetic peripheral neuropathy (Nakhjavani et al., 2010).

### Poly ADP-Ribose Polymerase

Poly ADP-ribose polymerase-1(PARP-1) is a ribozyme with many regulatory functions and rich content. It can repair the DNA, maintain genome integrity and regulate the expression of many proteins, apoptosis, and death at the transcriptional level. Transplantation of bone marrow from poly ADP-ribose polymerase (PARP)-/- mice into wild type (WT) mice could inhibit the occurrence of DPN. On the contrary, when reversed (bone marrow from WT mice was transplanted into PARP mice), PARP^−/−^ mice were vulnerable to DPN. *In vitro* experiments supported these results: when WT mice BM-derived cells and DRG were co-cultured in a high glucose environment, this led to the fusion of the two cells. This did not occur in a low glucose environment nor fuse with bone marrow-derived cells from PARP-/- mice (Terashima et al., 2012). PARP activation plays a vital role in the pathogenesis of diabetes and its complications. Activation of PARP in diabetic nerves can cause energy failure through vascular and non-vascular mechanisms. The activation of PARP can lead to the shortage of nutrient blood flow and sensory and motor conduction velocity in the neuro intima and lead to the degeneration of large and small nerve fibers. The motor nerve conduction velocity and the formation of sensory nerve conduction need PARP activation. Motor nerve conduction velocity and sensory nerve conduction function were less damaged in PARP deficient diabetic mice (Tesfaye and Selvarajah, 2012). Additional evidence found that the use of PARP inhibitor treatment in experimental diabetic patients could reduce the slowing of MCV and SCV and prevent the decrease of axon diameter and myelin sheath thickness. It is worth noting that inhibition of PARP prevents the increase of TNF-α and nitrotyrosine in the sciatic nerve and spinal cord (Drel et al., 2010).

Long term exposure to glucose leads to nonenzymatic glycosylation of peripheral myelin, which leads to further changes in the interaction between macrophages and myelin. It is worth noting that macrophages recognize the newly formed age products in the myelin sheath and internalize and attack it (Vlassara et al., 1984). Bekircan-Kurt et al. found that Rage was observed in distal skin biopsies of DPN patients, and the number of T cells adhering to blood vessels increased (Bekir Ca N-Kurt et al., 2015). In addition, when RAGE was deleted from diabetic male mice, it had a partial protective effect on the early electrophysiological parameters of chronic DPN. In contrast, insulin treatment had little impact on diabetic mice (De et al., 2017). IL-6 has a therapeutic effect on DPN. Giving IL-6 1 or 3 times a week had a significant preventive effect on decreasing MCV and SCV in STZ induced diabetic rats. It also improves heat sensitivity, reduced abnormal nerve fiber’s proportion, and prevents myelin thinning (Andriambeloson et al., 2010). Studies showed that AGE/RAGE signal led to the increase of NF-κB and IL-6 in DPN (Bierhaus et al., 2004). Advanced glycation end products (AGE) may accumulate naturally during aging. Interestingly, soluble RAGE therapy and RAGE knockout resulted in a decrease in NF-κB (Ravichandran et al., 2005; Figure 2).

### NLRP3

NLRP3 belongs to the pattern recognition receptor of nucleotide-binding oligomerization domain-like receptors (NLR). NLRs have nucleotide-binding oligomerization domain (Nacht), which is supplemented by C-terminal leucine-rich repeats (LRRs structure) and N-terminal caspase (card) or pyrin domain. The function of LRRs is ligand sensing and self-regulation, while card and PYD structures regulate the interaction of downstream signal hemoproteins. Nacht structure is the only typical structure of all NLR families and is activated by ATP-dependent oligomerization. NLRPs subgroup includes PYD and LRR, and NLRP3 is a part of the NLRs subgroup. NLRP3 inflammasome is the most characteristic inflammasome in the NLRs. It comprises NLRP3, autophagy-related spotted protein (ACS), and cysteine aspartate specific protease caspase-1. Some scholars believe that the NLRP3 inflammatory body is a receptor of metabolic risk. NLRP3 inflammatory bodies play an important role in many non-infectious inflammatory diseases, such as gout, atherosclerosis, and diabetes. LRP3 inflammasome can activate caspase-1, then cut the precursor forms of inflammatory factors such as in IL-1β and IL-18, mature them and release them outside the cell, causing inflammatory response. IL-1β, reactive oxygen species (ROS), and thioredoxin interacting proteins are associated with the pathogenesis of type 2 diabetes. Sustained hyperglycemia induces ROS. ROS directly activates NLRP3 inflammasome or indirectly activates NLRP3 inflammasome by stimulating thioredoxin to release thioredoxin interacting protein. This process further aggravates chronic hyperglycemia and worsens diabetes. NLRP3 activation is the central link in the inflammatory mechanism of diabetes.

## Immune Cells and Diabetic Peripheral Neuropathy

### Lymphocytes

In the pathogenesis of DPN, the immune mechanism should not be ignored. The cellular immune mechanism with regulatory T lymphocyte subsets is the most important, which participates in the negative regulation mechanism of immune-mediated inflammation.

The cytotoxicity of CD8^+^ T lymphocytes to Schwann cells is also involved in the development of DPN. A quantitative immunohistochemical study was carried out on 20 cases of DPN sural nerve biopsy specimens to determine whether there was the infiltration of neural and epicardial lymphocytes in the diabetic nerve. There were 129 CD3^+^ cells in each tissue section of DPN patients and 0–5 cells in normal control. Diabetic nerve T cells infiltrated mainly CD8^+^ cells. The activated lymphocytes expressed immunoreactive cytokines and primary histocompatibility class II antigens. Infiltrating T cells may participate in the pathogenesis of diabetic neuropathy through various mechanisms (Younger et al., 2015).

Myelin protein correlated with the insulin receptors. After insulin treatment, the expression of myelin protein is up-regulated, which alleviates the nerve damage. It indirectly reflects the down-regulation of the insulin receptor, up-regulation of myelin associated glycoprotein, and down-regulation of myelin basic protein, which directly inhibited the myelin sheath formation, thus causing damage to DPN (Younger et al., 2015).

In addition, the number of pericytes is also involved in immune regulation, which is inversely proportional to CD4^+^, CD8^+^ T lymphocytes. In mice models lacking pericyte, the immune-related cells reduced, indirectly confirming that the peripheral cells participate in the mediated immunity (Hong et al., 2015).

### Microglia

As innate immune cells, microglia can clear cell debris and foreign bodies and play the critical role of immune monitoring. Hyperglycemia and reactive oxygen species can also affect the local microenvironment of spinal cord and activate microglia. In turn, activated microglia synthesize and released inflammatory cytokines and neuroactive molecules, which induce the spinal cord injury-sensitive neurons (Couture et al., 2014). Activated microglia release various neuromodulators and neuroactive substances, such as reactive oxygen species, nitric oxide, peroxynitrite, prostaglandins, and pro-inflammatory cytokines, involved in hyperalgesia neuropathic pain of DPN. The expression of IL-1β and TNF-α in the spinal cord of STZ diabetic rats increased. The increase of IL-1β and TNF-α, as well as the thermal and mechanical hypersensitivity of rats, were inhibited by systemic or spinal administration of flucytosine, a non-selective metabolic inhibitor of glial cells, or minocycline, a selective microglia inhibitor. Systemic or spinal administration of flucytosine or minocycline inhibited IL-1 β And TNF- α As well as thermal and mechanical allergies in rats. A recent study found that atommoxetine (a new effective 5-hydroxytryptamine and norepinephrine reuptake inhibitor) had a sustained analgesic effect on STZ induced diabetic rats and could improve their depressive behavior. It could inhibit the activation of microglia and the phosphorylation of p38 and c-Jun N-terminal kinase (JNK) and reduce the inflammatory cytokines (Zhang et al., 2018).

### Macrophages

Due to the reports of many macrophages in DPN, some researchers used these immune cells as a means of treatment. Macrophages have two polarization states: M1 and M2 macrophages, which express many inflammatory factors such as inducible nitric oxide synthase (iNOS), IL-1β, TNF-α, and anti-inflammatory cytokines such as Arg-1 (Huo et al., 2018), respectively. Under high glucose, M1 macrophages and other immune cells were activated to express a large number of inflammatory factors, which led to Schwann cell apoptosis and the occurrence of PDPN. Studies showed that the inhibition of TNF- α, and the release of M1 and macrophages into M2 macrophages could induce the gradual recovery of nerve conduction velocity, nerve blood flow, and axonal morphology in streptomycin -induced diabetic rats (Omi et al., 2017).

Macrophages can alleviate some symptoms of DPN in STZ induced diabetic rats. The researchers used liposomes encapsulated chlorondronate and found that compared with untreated diabetic rats, treatment resulted in lower blood glucose levels, higher serum insulin levels, and reduced mechanical hyperalgesia. The treatment did not affect heat sensitivity and body weight (Mert et al., 2009). Strangely, when liposome-encapsulated chlorophosphonate was used in combination with the pulsed magnetic field, the treatment effect was insufficient, while each treatment used alone affected DPN symptoms (Tufan et al., 2013).

### Schwann Cells

Mitochondrial dysfunction in Schwann cells leads to lipid oxidation, early consumption of myelin, and accumulation of acylcarnitine lipid intermediates, leading to axonal degeneration and neuropathy (Viader et al., 2013). In addition, human Schwann cells exposed to high glucose can reduce the synthesis of phospholipids, which can be improved by aldose reductase inhibitors, suggesting that high glucose promotes the dyslipidemia of Schwann cells (Gonçalves et al., 2017). One study showed that glucose-stimulated Schwann cells to produce chemokines CXCchemokineligand-9(CXCL-9), CXCL-10, and CXCL-11 could induce the aggregation of T cells into diabetic neuropathy patients, thus promoting the development of neuropathy. These results also support that Schwann cells may be involved in the development of painful DPN (Tang et al., 2013).

## Therapy of Diabetic Peripheral Neuropathy

### First-Line Therapy

Tricyclic antidepressants include imipramine and amitriptyline, desipramine and nortriptyline. These blocked the reuptake of norepinephrine and serotonin by presynaptic neurons. Another mechanism that may contribute to its analgesic effect is to secure the uptake of 5HT and norepinephrine. A case control study showed that tricyclic antidepressive agents (TCAS) was influential in the treatment of DPN (Finnerup et al., 2005; Brouwers et al., 2010). Tricyclic antidepressants are well absorbed by oral administration, and their lipophilicity makes them widely distributed and easy to penetrate the central nervous system (CNS). However, due to the first pass metabolic changes in the liver, the bioavailability of tricyclic antidepressants is inconsistent. It needs to be increased to an effective dose, which must be administered continuously for 6–8 weeks (Max et al., 1992; Finnerup et al., 2010; Rudroju et al., 2013; Singh, 2014). Side effects include dry mouth, orthostatic hypotension, constipation, and urinary retention. In addition, the contraindications of TCAS are glaucoma and prostatic hypertrophy (Gandarias et al., 1998; Benbouzid et al., 2008).

Serotonin and noradrenaline reuptake inhibitors: Simultaneous inhibition of norepinephrine and serotonin reuptake can reduce DPN-related pain (Iqbal et al., 2018). We searched 13 studies showing the effect of serotonin and norepinephrine reuptake inhibitor (SNRI) on DPN. Duloxetine is the most studied SNRI. Eight randomized controlled trials showed that duloxetine was effective in the treatment of DPN (Goldstein et al., 2005; Wernicke et al., 2006; Raskin et al., 2006; Beard, Mccrink et al., 2008, Ajay D.; Wasan 2009; Joel et al., 2010; Tanenberg et al., 2011; Boyle et al., 2012; Lunn et al., 2014).

Calcium channel a2-δ ligands: Pain is one of the main symptoms of diabetic peripheral neuropathy, which seriously reduces patient’s quality of life. Studies showed that the first-line drug for alleviating diabetic peripheral neuropathic pain was calcium channel a2-δl, a modulator. Commonly used drugs are pregabalin and Gaba Martin. These drugs reduce the influx of neurons Na+ and Ca2+ and indirectly enhance the inhibition of γ-aminobutyric acid (GABA). It can reduce the activity of the N-methyl-D-aspartate receptor (NMDA receptor) by consuming the storage of excitatory neurotransmitter glutamate or blocking the active site of glutamate (Decroli et al., 2019).

### Second-Line Therapy

Opioid analgesics can be used as the first-line clinical treatment, but due to their safety and potential abuse, many guidelines consider opioids as the second-line treatment (Finnerup et al., 2010). Some studies have shown that strong opioids play a positive role in the treatment of peripheral neuropathy (Huse et al., 2001; Raja et al., 2002; Morley et al., 2003).

Tramadol is a weak opioid, which can inhibit the reuptake of norepinephrine and serotonin. Most international guidelines consider tramadol as second-line therapy (Boureau et al., 2003; Duhmke et al., 2006).

### Immunoinflammatory Therapy

In animal experiments, many methods were used to treat DPN. The combination of insulin and curcumin or resveratrol in STZ induced diabetic mice reduced the TNF-α and hypersensitivity *in vivo*, which was more evident than insulin or the use of two drugs alone. Thakur et al. (Thakur et al., 2016) also used virus-mediated IL-10 to reduce the mechanical and thermal hypersensitivity of STZ induced diabetic rats. This treatment prevented the increase of Toll-like receptors4 (TLR4), IL-1, phosphorylated p38, and phosphorylated protein kinase C levels and decreased NeuN and HSP70 protein levels in the diabetic control group. It is also noteworthy that IL-10 treatment prevented the activation of macrophages in DRG. Under hyperglycemic conditions, cultured DRGs exposed to TLR4 antagonists could avoid increasing protein TNF-α and showed a significant increase of HSP70. *In vivo* tests, diabetic animals exposed to TLR4 antagonists reduced TLR4 and TNF-α levels and increased mechanical sensitivity but did not increase heat sensitivity.

Ni et al., 2017) studied the effect of salidroside. Salidroside had anti-inflammatory properties. When salidroside treated DPN rats, they found that it decreased mechanical sensitivity only after 8 weeks of treatment and decreased thermal sensitivity until 8 weeks after 5 weeks. They also found that sensory nerve conduction velocity improved after 5 weeks of treatment. In terms of mechanism, the authors found that treatment could reduce the levels of pro-inflammatory cytokines TNF-α and IL-1 in the spinal cord and sciatic nerve. The study also found that treatment could reduce the protein expression of the P2X7 receptor in the spinal cord. P2X7 receptor played a role in mediating neuropathic pain by secreting pro-inflammatory factors.

### Molecular Therapy

Virus vector technology: Viral vector is the most efficient vector for gene transmission. Except for adenovirus, other viral vectors can integrate foreign genes into chromosomes by infecting host cells, which can be used in cells that are difficult to transfect. After the viral DNA was integrated into the host cell genome, it could express stable gene therapy potential (Liu et al., 2020). Lentivirus (LV) belongs to Retroviridae and is a diploid RNA virus. Lentiviral vectors are vectors based on the genome of lentivirus, in which multiple sequence structures related to virus activity are removed to ensure biological safety. Then exogenous genes are introduced into the genome skeleton. Tasyurek et al. injected LV carrying human glucagon-like peptide-1 (GLP-1) gene, which could reduce the blood glucose level of the T2DM rat model induced by a high-fat diet combined with streptozotocin, and its triglyceride level returns to normal. These results showed that LV could effectively transfer potentially therapeutic genes into islet cells for DPN treatment (Tasyurek et al., 2018). Adenovirus vectors (AdV) can infect a variety of human tissue cells. AdV has the advantages of high titer, strong gene transfection ability, non-integration into the host genome, and will not affect islet transplantation. At present, it has been widely used in gene transduction *in vitro*, vaccination *in vivo*, and gene therapy (Jane and Bradbury, 2003). In the treatment of diabetes, AdV is still in animal experiments. Suzuki et al. (Suzuki et al., 2004)used AdV to introduce insulin receptor substrate-2 (IRS-2) gene into IRS-2 deficient mice and found that the blood glucose level of mice returned to normal. The transcription factor pancreaticoduodenal homeobox-1 gene can be transfected into mouse islet cells by AdV to participate in islet maturation. In addition, the pancreatic differentiation transcription factor neurogenin 3 (Ngn3) could be transfected into mouse hepatocytes by adv to secrete insulin (Reach, 2001).

Stem cell therapy: Stem cells can differentiate into tissues such as fat, bone, and cartilage. They have strong self-renewal ability, multidirectional differentiation potential, and secrete a variety of cytokines. They have a good application prospect in the treatment of DPN. Mesenchymal stem cells (MSCs) therapy can improve the blood glucose level of diabetic patients from the vital point of diabetes. Bone marrow MSCs and pancreatic MSCs can be differentiated into Islet β Cells under the regulation of multiple growth factors and hormones. Then the blood glucose concentration of diabetic mice could be reduced by increasing secretion of insulin and C peptides (Lin et al., 2010; Mu and Li 2015). Transplantation of MSCs can promote the proliferation of islet cells, increase the number of insulin-secreting cells, improve the structure of islets, increase the synthesis and secretion of insulin, and reduce blood glucose concentration.

### Non-drug Therapy

Exercise: Exercise plays an irreplaceable role in the treatment of diseases. It is the basis of therapy and should run through the whole process of DPN treatment. Exercise therapy mainly includes aerobic exercise and anti-resistance exercise. Anti-resistance exercise has a better therapeutic effect on DPN. Kluding et al. intervened 17 DPN patients with moderate intensity, aerobic and anti-resistance exercise under the guidance and supervision of professional coaches. After 10 weeks of intervention, they carried out self-control. The research results showed that exercise could alleviate DPN patient’s pain symptoms, increase the density of nerve fibers in the epidermis, and improve their neurological symptoms. Exercise can also protect the nerve injury of DPN patients by enhancing the ability of nerve regeneration (Kluding et al., 2012; Singleton et al., 2014). In terms of optimizing the exercise therapy program, we can work out a safe and effective individualized exercise program through multidisciplinary cooperation.

Electrotherapy: In recent years, more and more scholars have pay attention to electrotherapy, including transcutaneous electrical nerve stimulation (TENS); Peripheral nerve, nerve root, spinal cord, deep brain, and epidural motor cortex stimulation; Pulsed magnetic field and static magnetic field; High-frequency external muscle stimulation (Pieber et al., 2010). However, among all electrotherapy measures, only tens are recommended by the American Academy of Neurology (AAN) for pain treatment of DPN (Dubinsky and Miyasaki, 2010). Upton GA et al. discussed the effects of two different intensities of TENS on patients with DPN. The results showed that the acupuncture-like stimulation mode of 2 Hz and 200 ms could better alleviate the pain caused by DPN without adverse reactions (Zotz and De, 2015). Current studies believe that the theoretical basis of electrical stimulation improves the microcirculation of DPN patients and increases the nerve blood flow to alleviate the pain caused by ischemia. However, Gossrau g et al. conducted a randomized placebo-controlled experiment. The results showed that the therapeutic effect of TENS was not better than that of the placebo group, and the difference was not statistically significant (Gudrun et al., 2011). TENS has the advantages of safety, noninvasive, simple operation, and good compliance. However, its exact role is still controversial, which may be related to the small sample size of existing studies, short intervention cycle, unclear long-term efficacy, and lack of evidence-based basis, limiting its clinical application.

Acupuncture: Acupuncture and moxibustion can improve nerve conduction and alleviate patient’s clinical symptoms with DPN. The mechanism may be related to acupuncture, and moxibustion can improve microcirculation and reduce ischemia and hypoxia of nerve tissue. Jeon et al. treated 9 outpatients with painful DPN with acupuncture and moxibustion for 4 weeks. The results showed that acupuncture and moxibustion could significantly improve the pain symptoms of patients. They believed that acupuncture and moxibustion were effective but did not study their mechanism (Jeon et al., 2014). The relevant systematic evaluation analyzed the effect of acupuncture and moxibustion on DPN. Although the results showed that the outcome was better than conventional treatment, it also pointed out that the development of acupuncture and moxibustion on DPN was uncertain due to the low quality of relevant RCT experimental literature included in the study (Dimitrova et al., 2017).

### Complications and Comorbidities Therapy

Most patients with diabetic peripheral neuropathy are accompanied by depression, anxiety, insomnia, poor appetite, lower body weight, suicidal ideation, drug abuse. These complications affect their mental health and have adverse effects on the treatment and rehabilitation of their patients, which is not conducive to their physical health. Compared with patients with uncomplicated diabetes mellitus, diabetic peripheral neuropathy patients have more severe pain, and the severity of negative emotions and comorbidities is more serious. They can be treated with first-line drugs for DPN, symptomatic pain relief, and relief of negative emotions.

In addition, for patients with diabetic peripheral neuropathy, clinical targeted psychological intervention should be carried out to alleviate patient’s negative emotions. Psychological intervention should be based on different causes of anxiety and depression, including cognitive intervention, environmental intervention, and emotional intervention. Particular attention should be paid to patient’s family and social intervention because family warmth, social understanding, and support are good drugs for treating psychosomatic diseases.

## Conclusion

In summary, diabetic peripheral neuropathy is related to many factors, such as metabolism, immunity, heredity, etc., the etiology is currently uncertain. In this review, we highlighted the latest immunological mechanism of DPN and explored their corresponding potential therapeutic targets. Its complex mechanism makes it difficult to treat. There is no specific drug therapy. With the increase of diabetic patients, more and more patients suffer from the complications of diabetes. This makes the requirement to control diabetes, and its complications become more urgent. The pathogenesis of diabetic peripheral neuropathy needs to be further in-depth and comprehensive research to achieve more standardized management of diabetic peripheral neuropathy.
